# THE PAUSE TRIAL: LESSONS ABOUT SURGICAL PAUSE FROM AN RCT

**DOI:** 10.1093/geroni/igae098.1563

**Published:** 2024-12-31

**Authors:** Shipra Arya

**Affiliations:** Stanford University, Stanford, California, United States

## Abstract

Effective clinical care pathways that integrate preoperative frailty assessment and management to optimize high-risk older adults undergoing surgery are urgently needed to improve their outcomes. We designed a hybrid1 effectiveness implementation step-wedge cluster randomized trial called the Patient-Centered mUltidiSciplinary care for vEterans undergoing surgery (PAUSE) to test frailty screening and referral to a multidisciplinary member board to guide optimization of surgical patients prior to surgery. The PAUSE trial includes Veterans scheduled for elective surgery at 3 tertiary care VAMCs (Palo Alto, Houston, Nashville) and 7 specialties in each center (general, vascular, orthopedics, cardiothoracic, urology, neurosurgery and plastics/ENT). The intervention is referral to a “multidisciplinary PAUSE board” (e.g., surgery, anesthesia, geriatrics, palliative care, case management, rehabilitation and nutrition) for recommendations after frailty screening. Each ‘step’ was a randomly chosen specialty group transitioning from usual care to the PAUSE intervention. The Consolidated Framework for Implementation Research was used to guide mixed methods formative evaluation and analysis of factors that influence implementation. Key stakeholders identified champions, potential barriers and facilitators, possible intervention adaptations, and indicated limited time, staff shortage, and the concern that the PAUSE board may delay surgery as main barriers to implementation. Nurses were identified as partners in intervention execution. Surgical and nursing leadership engagement was indicated as crucial to adopting this new healthcare model. In conclusion, formative evaluation delivers timely and actionable findings to inform the intervention and identify implementation modifications to optimize opportunities for success. Multistakeholder involvement and accounting for clusters and sites variations is important for successful implementation.

